# Immunomodulatory Effect and Biological Significance of β-Glucans

**DOI:** 10.3390/pharmaceutics15061615

**Published:** 2023-05-29

**Authors:** Xuemei Zhong, Guoqing Wang, Fu Li, Sixian Fang, Siai Zhou, Akihiro Ishiwata, Alexander G. Tonevitsky, Maxim Shkurnikov, Hui Cai, Feiqing Ding

**Affiliations:** 1 School of Pharmaceutical Sciences (Shenzhen), Shenzhen Campus, Sun Yat-sen University, Shenzhen 518107, China; 2 Medical College, Shaoguan University, Shaoguan 512026, China; 3 The Eighth Affiliated Hospital, Sun Yat-sen University, Shenzhen 518033, China; 4 RIKEN Cluster for Pioneering Research, Wako 351-0198, Saitama, Japan; aishiwa@riken.jp; 5 Faculty of Biology and Biotechnology, National Research University Higher School of Economics, Moscow 117418, Russia

**Keywords:** β-glucans, immune system, dendritic cells, adjuvants

## Abstract

β-glucan, one of the homopolysaccharides composed of D-glucose, exists widely in cereals and microorganisms and possesses various biological activities, including anti-inflammatory, antioxidant, and anti-tumor properties. More recently, there has been mounting proof that β-glucan functions as a physiologically active “biological response modulator (BRM)”, promoting dendritic cell maturation, cytokine secretion, and regulating adaptive immune responses—all of which are directly connected with β-glucan-regulated glucan receptors. This review focuses on the sources, structures, immune regulation, and receptor recognition mechanisms of β-glucan.

## 1. Introduction

β-glucan is a kind of polysaccharide with multiple physiological functions and is known as a “biological response regulator” because of its multiple biological functions [1]. The first defensive line of body immunity is innate immunity. In its early stage, it mainly uses phagocytic cells such as macrophages and neutrophils to engulf and kill pathogens invading the body and then further activates the adaptive immune system by secreting cytokines and chemokines. β-glucan has been found to affect several types of immune cells, including macrophages, natural killer cells, and neutrophils, resulting in various immunological effects. In recent decades, tumor immunotherapy has made extensive use of β-glucan as a natural biological effect regulator [2]. Current clinical applications of β-glucans include yeast, lentinan, Coriolus versicolor polysaccharide, mycobacterium polysaccharide, and oat. The different types of β-glucan influence the strength and immune response, depending on the source, structure, water solubility, and molecular weight [3]. The body recognizes invading pathogenic microorganisms through the pattern recognition receptor (PRR) and initiates the body’s immune response to pathogens through a series of biochemical reactions. Currently, the following β-glucan receptors have been identified: dendritic cell (DC)-associated C-type lectin-1 (Dectin-1) [4], complement receptor 3 (CR3), cluster of differentiation 11b (CD11b)/CD18, αMβ2-integrin, macrophage differentiation antigen-1 (Mac-1) [5,6], lactosylceramide (LacCer) [7], and scavenger receptors (SRs) [8]. In this review, the source and structure, immunoregulation, and receptor recognition mechanism of β-glucan are discussed, which offers fresh perspectives on the development of natural immune enhancers for anti-tumor immunotherapy.

## 2. β-Glucan Sources and Properties

**Figure 1:**
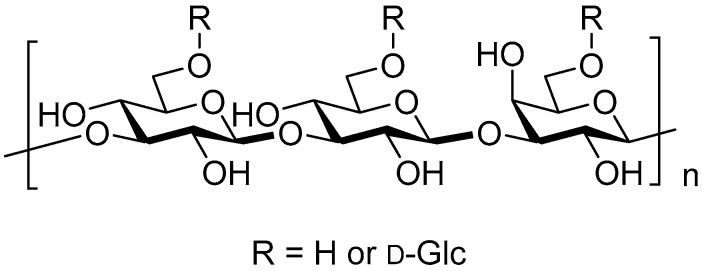
Example of (1→3)-β-D–glucan.

Glucan is a homopolysaccharide of glucose as a monomer structure. It is the most common polysaccharide in nature, widely distributed in bacteria, fungi, and other plants, consisting of two types depending on the difference in the stereoisomer of the glycosidic bond as α- and β-linked glucans. The α- and β-glucans are well known to be the energy source of the body and the indigestible fibers with obvious physiological functions [9,10,11,12,13,14,15,16] mainly as α- and β-(1→4)-linked liner structures between glucose residues (amylose and cellulose), respectively. The latter one, β-glucan, has been attracting attention in recent years as an antineoplastic immunostimulant that mainly comes from yeast, barley, oats, fungi, mushrooms, and algae [17,18,19,20]. β-Glucan is also a component of the cell wall of certain pathogenic fungal (*Pneumocystis carinii*, *Cryptococcus neoformans*, *Aspergillus fumigatus*, *Histoplasma capsulatum*, *Candida albicans*) and fungi (*Saccharomyces cerevisiae*) [21,22]. The cell wall of fungi is mainly composed of polysaccharides and glycoproteins. For example, the cell wall of *S. cerevisiae* consists of three layers: the inner layer is insoluble β-glucan (30–35%); the middle layer is soluble β-glucan (20–22%); the outer layer is glycoprotein (30%) [23]. The β-glucan mainly exists in nature in the form of liner or branched chains of (1→2)-, (1→3)-, (1→4)-, and (1→6)-β-glucan [24,25] (Table 1, Figure 1). In addition to abundant (1→4)-β-glucan in plant cellulose, plant hemicellulose includes linear β-glucans with (1→3)-(1→4)-mixed linkages, containing tri-and tetrasaccharide (1→4)-β-glucan fragments inserted in random order. In this case, the contents of β-(1→4) and β-(1→3) linkages in the β-glucans are approximately 70% and 30%, respectively. Lichenan (Lichenin) from Icelandic moss consists of a linear (1→3)-(1→4)-mixed linkages with [→4)-β-glycosyl-(1→3)-β-glycosyl-(1→] repeating unit. Barley and oat glucans from *Hordeum vulgare* and *Avena sativa*, respectively, consist of linear (1→3)-(1→4)-mixed linkages as well. *Agrobacterium tumefaciens* produces a β-(1→2)-glucan structure whose function has not been reported although cyclic β-(1→2)-glucan plays an important role for plant pathogen in evading the host immune system [26,27,28,29]. Except for the large quantity of plant β-(1→4)-glucan and very rare (1→2)-β-glucan as its cyclic forms, different sources of β-glucan from algae and bacteria have certain differences in structure and function, whose glucan structures are mainly a linear (1→3)-β-glucan. Yeast produces mainly (1→3)-β-glucan containing β-(1→6)-branches. β-glucan of the fungal origin mainly contains a linear structure with a combination of β-(1→3) and β-(1→6) linkages, and cereal-derived one has a linear combination of β-(1→3)- and β-(1→4)-linkages [25].

**Figure 2:**
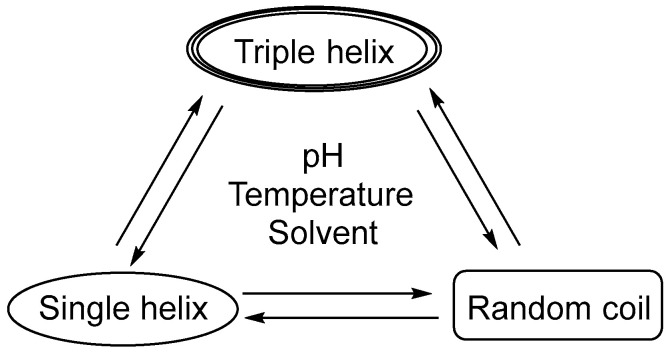
Conformational change in secondary structures of (1→3)-β-glucans.

β-glucan, mostly referred to as (1→3)-β-glucan, can be divided into the single helix, triple helix, or random helix (irregular helix) according to its three-dimensional conformation [41]. The three structural formulas can be transformed into each other (Figure 2), for example, the triple helix is opened to form a single helix structure under alkaline conditions such as NaOH [42,43]. Meanwhile, the single helix also can become an irregular helix, which is restored to a triple helix structure under heating or dialysis conditions. It is shown that the three-dimensional structure of the (1→3)-β-glucan polymers is an important determinant of receptor–ligand interactions [8]. Insoluble particulate (1→3)-β-glucan is thought to activate DCs and macrophages in rats through the Dectin-1 pathway. This activation is believed to be enhanced depending on the degree of molecular polymerization and the content of the β-glycoside bond [44]. While water-soluble (1→3)-β-glucan can bind to these cells, it does not activate them [45]. Furthermore, (1→3)-β-Glucan has a wide range of physiological functions [46], including immune system enhancement [47,48], anti-tumor [49], anti-infection [17], anti-radiation [50], metabolism regulation [51,52], anti-inflammatory [53], antioxidant [54], hypoglycemic [55], and reductions in serum lipids [56]. Additionally, it has been used as an additive in the beauty and skincare industries due to its anti-radiation, anti-aging, and free radical scavenging properties. Furthermore, (1→3)-β-glucan can help to reduce body absorption and level of cholesterol and other blood lipids in blood and has been utilized in the food and healthcare industries [57].

## 3. Immunostimulatory Properties of (1→3)-β-Glucan

As a natural barrier of the human body, the immune system has the functions of immune surveillance, defense, and regulation. It can be divided into adaptive immunity and innate immunity. The former is subdivided into cellular immunity and humoral immunity, which are respectively exerted by T lymphocytes and B lymphocytes, and the latter is mainly exerted by innate immune cells, such as monocyte macrophages and natural killer (NK) cells [58]. Therefore, modern medicine mainly evaluates immune function from four aspects: cellular immunity, humoral immunity, mononuclear macrophage phagocytosis, and NK cell activity. Carbohydrates are common surface molecules in biological systems. Due to their rich structural diversity, carbohydrate molecules play an important role in cell recognition and signal transduction, including immune recognition and activation [59,60]. Furthermore, (1→3)-β-glucan is a polysaccharide adjuvant widely existing in bacterial and fungal cell walls, which can stimulate antibacterial immune response [60]. In the 1950s, Dr. Pillemer first discovered and reported that there was a substance in the yeast cell wall that could improve immunity [61]. In later research, Diluzio’s group discovered that the immunity-boosting substance in the yeast cell wall was (1→3)-β-glucan, isolated from baker’s yeast [62,63].

Yeast (1→3)-β-glucan activates various immune cells, including macrophages and neutrophils, leading to increased production of interleukin (IL), cytokinin, and special antibodies. This comprehensive stimulation of the immune system prepares the body to better fight against diseases [64,65]. In addition, yeast (1→3)-β-glucan restores the ability of lymphocytes to produce cytokines such as IL-1 and effectively regulates immune function [66,67,68]. Many experiments have indicated that yeast (1→3)-β-glucan promotes the production of IgM antibodies, improving humoral immunity. Moreover, yeast (1→3)-β-glucan activates toll-like receptor 2 (TLR2), inducing nuclear factor (NF)- κB activation and tumor necrosis factor (TNF)-α secretion, as well as regulatory antigen-presenting cells and immune tolerance [69,70]. The yeast (1→3)-β-glucan-activated cells stimulate the host’s non-specific defense mechanism and are thus being studied for their potential in cancer, infectious disease, and wound treatment. In 2008, β-glucan extracted from *S. cerevisiae*’s yeast was released by the US Food and Drug Administration (FDA) as a safe food ingredient that can be added to general food. It is a very rare active immune substance, which can kill harmful viruses and maintain good immunity. Many years ago, National Aeronautics and Space Administration (NASA) listed yeast glucan as a food for astronauts to enhance their immunity.

Many researchers have not only demonstrated the regulatory effect of yeast (1→3)-β-glucan on immunity, such as induction of autoimmune arthritis or enhancement of nitric oxide (NO) synthesis, through in vitro cell experiments or in vivo experiments in mice, respectively [71,72,73], but also showed that β-glucan also has the effect of immune stimulation on zebrafish [74,75].

Wu et al. reported that the addition of (1→3)-β-glucan can also lessen the inflammatory response after lipopolysaccharide (LPS) stress [76]. Yeast (1→3)-β-glucan is also an important enhancer of mucosal immunity in the digestive tract [77]. The digestive system is the primary point of contact for many pathogens and foreign substances, and mucosal immunity in the system plays a vital role in defending against these threats. Additionally, it has also been found that (1→3)-β-glucan can improve the level of lysozyme in animal serum and the antibody titer [78]. Yeast (1→3)-β-glucan can activate neutrophils and phagocytes in gastrointestinal tissues, thereby further activating and affecting the “immune nerve endocrine” regulatory network, enhancing its anti-infection, anti-stress, and cellular adaptive protection capabilities and also enhancing macrophage-mediated tissue repair, accelerating the repair process of ulcers, and improving the repair quality [79]. Yeast β-glucan has the ability to bind with surface receptors of macrophages, neutrophils, and lymphocytes, which can affect the cellular signaling process, activate the immune activity of lymphocytes, and enable them to swiftly reach the site of infection [80]. It acts as an immune response booster and facilitates whey protein. Whey protein is a high-quality protein that contains all of the essential amino acids needed by the body. Combining with β-glucan, it can activate immune cells. While whey protein provides the building blocks necessary for these cells to function properly, they can work synergistically to enhance the immune response [81]. The latest research found that pre-treatment of mice with (1→3)-β-glucan can reduce the growth of tumors and elucidated that β-glucan transcriptomically and epigenetically rewires granulopoiesis and reprograms neutrophils towards an anti-tumor phenotype to form a long-term innate immune memory, i.e., trained immunity. Moreover, the anti-tumor effects of (1→3)-β-glucan-induced trained immunity can be transferred to recipient initial mice via bone marrow transplantation [82]. Furthermore, (1→3)-β-glucan can stimulate the innate immunity of Pagrus auratus by enhancing the respiratory burst of macrophages [83]. Besides for humans, Chang et al. also showed that the addition of (1→3)-β-glucan to the diet of shrimp enhanced the bacteriophage activity of blood cells, cell adhesion, and production of reactive oxygen species [84].

**Figure 3:**
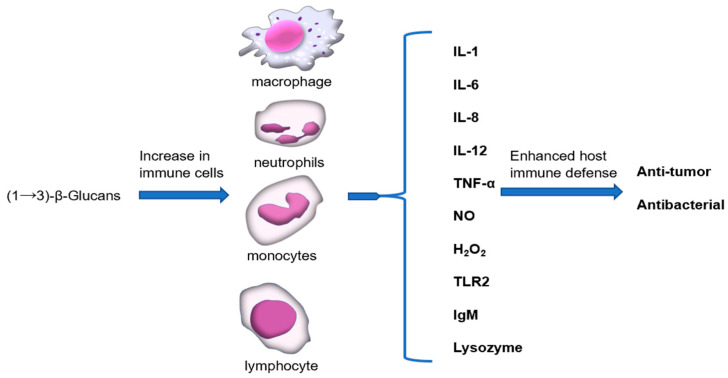
Immunomodulatory action of (1→3)-β-glucans.

Macrophages are essential to every stage of host defense and are engaged in both innate and adaptive immune responses in case of infection. The pathogen crosses the epithelial barrier, following phagocytosis by macrophages and digestion by lysosomal enzymes, which are important processes for presenting antigens from the pathogens as phagocytic activity, and lysosomal enzymes determine the function of macrophages [85]. The secretion of cytokines (IL-1, IL-6, IL-8, IL-12, TNF-a) and inflammatory mediators (NO, hydrogen peroxide (H_2_O_2_)) are also the downstream effect of these cells. Thus, β-glucan-activated macrophage function enhances host immune defense (Figure 3) [86,87]. Furthermore, (1→3)-β-Glucan is an effective immunomodulator [88,89,90], which can enhance the anti-tumor activity of peritoneal macrophages. In vitro studies showed that the killing effect of monocytes and neutrophils on microorganisms in healthy volunteers was enhanced after taking (1→3)-β-glucan. In addition to activating macrophages, T cells, and natural killer (NK) cells, (1→3)-β-glucan also activates complement components through a selective activation pathway. When (1→3)-β-glucan is present, it can bind to complement component C3, which triggers a cascade of reactions leading to the activation of the alternative pathway. This results in the formation of the C3 convertase enzyme, which cleaves C3 into iC3b. iC3b then binds to the surface of the pathogen, marking it for destruction by immune cells [5,6]. Activation of complement components through this pathway is important because it allows for a more targeted response to pathogens, without causing excessive inflammation or tissue damage. Vaccine adjuvants have a variety of mechanisms, typically including storage effects, promoting antigen presentation, increasing the secretion of immunomodulatory cytokines to control cellular responses with T and B, stimulating innate immunity, and indirectly modulating adaptive immune responses [91]. At present, (1→3)-β-glucan is an attractive candidate for immune adjuvants and is used in a wide range of vaccine development. It can activate the immune system and induce the Th1 immune response [92,93]. The potential effects of (1→3)-β-glucan as an adjuvant on the effectiveness of a severe acute respiratory syndrome coronavirus 2 (SARS-CoV-2) virus vaccination were discussed by Alfredo’s group (Figure 3) [94].

## 4. Immunoregulatory Receptor of (1→3)-β-Glucan

**Figure 4:**
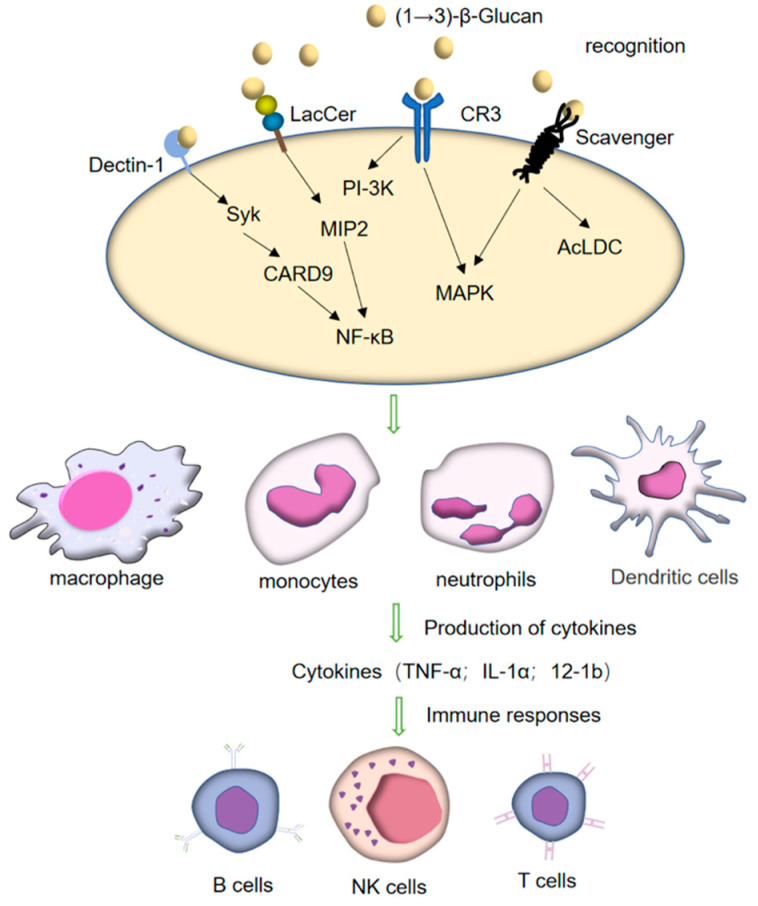
Immune activation induced by (1→3)-β-glucans: (1→3)-β-glucans can act on many receptors such as Dectin-1, CR3, LacCer, and scavenger found on the immune cells (monocytes, macrophages, DCs, and neutrophils).

The innate immune response triggers the immune system through the recognition and phagocytosis by phagocytes. Pathogen-related molecular pattern substances (PAMP_S_) activate the adaptive immune response process by recognizing and binding to the pattern recognition receptor (PRR) on the membrane structure of phagocytes. Most cell surface immune receptors, such as TLR_S_, nucleotide-binding and oligomerization domain (NOD)-like receptors (NLRs), and major histocompatibility complexes class I and class II (MHC-I and MHC-II), are glycoproteins. TLRs, NLRs, C-type lectin, and sialic acid-binding immunoglobulin (Ig)-like lectins (Siglecs), among other crucial receptors for immune cell activation, can identify sugar-containing ligands, including sugars expressed on the surface of many pathogenic microorganisms and cancer cells (Figure 4) [95]. As an important pathogen-associated molecular pattern (PAMP), (1→3)-β-glucan plays an immunomodulatory role mainly through three types of PRR: (1) Dectin-1 receptors; (2) CR3 [96]; (3) other receptors including scavenger receptor and LacCer [97] (Figure 4). It can be recognized by pattern-recognition receptors (PRRS) expressed on the surface of these innate immune cells, promoting the activation, maturation, and production of cytokines of immune cells, thus starting the innate immune response and regulating the subsequent adaptive immune response. Studies have shown that (1→3)-β-glucan can combine with C-type lectin Dectin-1 and CR3, promote the activation of secreted cytokines and B-cell T-cells, and enhance humoral and cellular immune responses [98]. Different sources, structures, and formulations of (1→3)-β-glucan can stimulate innate and adaptive immunity in different ways. In vitro and in vivo studies have shown that the molecular structure, molecular weight, and the number of branches are the key determinants of its immune activity [99].

Antigen presenting cells (APCs) can be divided into three types: DCs, macrophages, and B cells, these three types of cells are white blood cells and originate from bone marrow tissue [96]. DC-associated C-type lectin-1 (Dectin-1) is an important receptor of (1→3)-β-glucan. Much progress has been achieved since it was first found on the surface of DCs in the study of its anti-tumor mechanism [100,101,102]. The β-glucan receptor (β-GR) is made of three components: a C-type lectin-like carbohydrate recognition domain, a short stalk, and a cytoplasmic tail with a tyrosine-based immune receptor activation motif [100]. It recognizes carbohydrates containing β-(1→3)-linked and β-(1→6)-linked glucan bonds [103]. The Dectin-1 (β-GR) receptor is not restricted to dendritic cells but is broadly expressed, with the highest surface expression on myeloid cell populations (monocyte/macrophage and neutrophil lineages). β-GR is also expressed by dendritic cells and a subset of T cells, albeit with lower surface expression levels [104]. Many studies have demonstrated that after recognition with (1→3)-β-glucan, Dectin-1 can trigger its own intracellular signal transduction through the cytoplasmic immunoreceptor tyrosine-based activation motif (ITAM)-like motif, activating immune cells to produce a series of cellular reactions, such as phagocytosis and endocytosis of (1→3)-β-glucan, inducing respiratory burst, maturation of DCs, and producing various inflammatory cytokines TNF-α, IL-1a, IL-1b, IL-6, and chemokines, C-X-C motif chemokine ligand 2 (CXCL2), C-C motif chemokine ligand 3 (CCL3), and granulocyte-macrophage colony-stimulating factor (GM-CSF) [105,106,107]. Recruitment of spleen tyrosine kinase (Syk), activation of caspase recruitment domain family member 9 (CARD9), activation of NF-κB, mitogen-activated protein kinases (MAPKs), and activation of nuclear factor of activated T-cells (NFAT) are all components of the Dectin-1 downstream signal transduction pathway [108]. The phagocytosis of macrophages on non-conditioned microorganisms can trigger the host’s innate immune response against infection. Cytoplasmic phospholipase A2 (cPLA2) is activated in the process of phagocytosis, releasing arachidonic acid-producing biomass, causing acute inflammation. Dectin-1 receptor can also stimulate macrophages to release arachidonic acid and cyclooxygenase 2 (COX2) expression pattern recognition receptor through pathogenic yeast and yeast cell wall. Pure particulate (1→3)-β-glucan stimulates arachidonic acid release and enhanced COX2 expression macrophage-activated lipopeptide-2 (MALP-2) [109]. Dependent on TLR2, the first result on the synergistic effect of Dectin-1 and TLR2 to activate the proinflammatory response of macrophages to mycobacterial infection has been established [110]. The Dectin-1 receptor is a PRR widely expressed in macrophages and DCs. Furthermore, (1→3)-β-glucan is specifically recognized by the Dectin-1 receptor, of which the activation can also promote Th17 cell differentiation [111,112]. Although both soluble (1→3)-β-glucan and particulate β-glucan bind to Dectin-1, the downstream signal is only triggered by the latter [113], resulting in the release of tumor necrosis factor TNF-a, a marker of Dectin-1 activation [114]. Both (1→3)-β-glucan and (1→6)-β-glucan can effectively activate the bypass pathway of complement components, leading to fungal conditioning and the recruitment of inflammatory cells. The receptor Dectin-1 can stimulate the activation of Th1, Th17, and cytotoxic T-cell responses, reverse immune tolerance, and restore the secretion of cytokines [115,116].

The complement system is a kind of activated protein with enzyme activity, which widely exists in serum, tissue fluid, or cell surface. The complement system includes more than 30 kinds of soluble proteins and membrane-binding proteins, which can be divided into three categories according to their different biological functions: complement intrinsic components, complement regulatory proteins, and complement receptors (CR). The activated complement system has a precise regulatory mechanism, and the activated complement products have biological functions, such as cell lysis, regulating phagocytosis, clearing immune complexes, and mediating inflammatory reaction [117]. CR3, also called Mac-1, CD11b/CD18, or αMβ2 lectin, belongs to the family of leukocyte adhesion receptors as an important member [118]. It is present on the surface of macrophages, NK cells, B lymphocytes, cytotoxic T cells, and neutrophils and is also expressed on activated CD8+ T cell subsets and spleen DC membranes. By facilitating contact between effector cells and target cells it enhances phagocytosis. It is a membrane glycoprotein composed of two peptide chains with two distinct domains—one specifically bound to (1→3)-β-glucan and one particularly attached to the inactivated form of C3b fragment from serum C3 (iC3b). As a (1→3)-β-glucan receptor, CR3 has one or more exogenous lectin action sites distributed on its α-methylene. When (1→3)-β-glucan binds to these sites, they transmit signals to the cell to initiate the regulation of cytotoxins and phagocytic immune responses [2,119,120]. Michalek et al. reported that the leukocytes with activated CR3 on their surface have obvious killing power against tumor cells that form immune complexes on the cells [121], Ferreira and others found that (1→3)-β-glucan binds to CR3 on the surface of macrophages. It can activate phosphatidylinositol-3 kinase (PI3K) and MAPK signal pathways, promote the generation of inflammatory factors, and play its role in immune regulation [122]. The latest research shows that blocking CR3 can significantly lower the endocytosis of (1→3)-β-glucan by neutrophils and inhibit the production of (1→3)-β-glucan-induced reactive oxygen species [123]. By binding with CR3 on macrophages or NK cells, (1→3)-β-glucan continuously triggers the cytotoxicity of cells against iC3b tumor tissues and enhances the phagocytosis of macrophages and NK cells.

Other receptors include scavenger receptor, LacCer, etc. In the late 1970s, Goldstein and others first reported the binding site of acetylated low-density lipoprotein (AcLDL) on macrophages, which could mediate the uptake and degradation of AcLDL. The scavenger receptor (SR) is a glycoprotein that mainly exists on the surface of the macrophage membrane, can specifically bind and ingest oxidized low-density lipoprotein (ox-LDL), and has the function of binding with multiple ligands. It is a glycoprotein that induces the activation of urokinase-type plasminogen and the generation of inflammatory cytokines. NO is a crucial effector molecule for macrophage activation. Fucose and (1→3)-β-glucan were discovered to combine with scavenger receptor to play an immunomodulatory role [124]. Scavenger receptors play two roles in the process of the immune response: first, as pattern recognition receptors in the immune system to clear foreign bodies by recognizing specific pathogen-related molecular patterns; second, to clean up apoptotic nuclear fragments in vivo by identifying damage-related molecular patterns [125]; LacCer (CDw17) is a glycosphingolipid found in the plasma membrane of multiple kinds of cells. It was identified as (1→3)-β-glucan receptor through biochemical analysis of the interaction between β-glucan and isolated human leukocyte membrane components [126]. It has been demonstrated that the binding of (1→3)-β-glucan with this receptor can activate macrophage inflammatory protein (MIP)-2 and NF-κB, enhancing the oxidative burst and antibacterial function of neutrophils. However, the mechanism by which this occurs is yet unclear.

## 5. Clinical Applications of (1→3)-β-Glucans

(1→3)-β-Glucan has gained significant attention in recent years due to its potential health benefits. Numerous studies have been conducted for a long time to investigate the impact of (1→3)-β-glucan on various health conditions and have found many promising results. In Table 2, the clinical research on (1→3)-β-glucan and its immunomodulatory effects are summarized. Gudej et al. evaluated the efficacy of oat (1→3)-β-glucans in treating gastritis and found that oat β-glucans improved the quality of life for patients with gastritis, suggesting that it can be a potential natural treatment for gastritis and related gastrointestinal disorders [127]. Patients with high-risk neuroblastoma who had previously experienced disease progression showed strong antibody responses when treated with the GD2/GD3 vaccine in combination with (1→3)-β-glucan [128]. Medeiros et al. first studied to investigate the impact of *S. cerevisiae* (1→3)-β-glucan on venous ulcer healing in humans, and it could serve as a natural biological response modifier for wound healing [129]. Supplementing with 3 g/day of oat (1→3)-β-glucan can effectively reduce low density lipoprotein cholesterol (LDL-C), total cholesterol (TC), and non-high density lipoprotein cholesterol (HDL-C) levels in individuals with mild hypercholesterolemia over the medium term [130]. It was suggested that adding insoluble (1→3)-β-glucan from *Pleurotus ostreatus* to the diet may help regulate the exercise-induced alterations in NKCA observed in highly trained athletes [131]. Administration of yeast (1→3)-/(1→6)-β-glucan on a daily basis may provide protection against upper respiratory tract infections (URTIs) and shorten the duration of URTI symptoms in older individuals upon infections [132]. Furthermore, (1→3)-β-glucan has the potential to enhance serum IL-12 levels, shorten mechanical ventilation duration, and decrease organ failure in critically ill patients with multiple trauma [133]. Lehne et al. reported about soluble barley (1→3)-β-glucan (SBG) and observed an increase in the concentration of immunoglobulin (IG) A in saliva [134]. The preparation of yeast (1→3)-β-glucan increased the body’s ability to protect against invading pathogens [135]. Lee et al. and Carpenter et al. found that (1→3)-β-glucan has the potential to stimulate protective immunity without enhancing inflammation and modify immune responses after a strenuous exercise session, respectively [136,137]. However, while all (1→3)-β-glucans share a similar structure, the biological differences between (1→3)-β-glucans from different sources exist due to their differences in molecular weight, solubility, and purity as well as contents in other types of β-glucans with various branching patterns shown in Table 1. As shown in Table 2, (1→3)-β-glucans from different sources exhibit varying biological activities and clinical outcomes. Understanding these differences is crucial when evaluating the potential health benefits of β-glucans from different sources.

## 6. Conclusions

Among various β-glucans shown in this review, β-glucans possessing (1→3)-β-glucan backbone are a new, safe, and effective bioregulator. They exert a wide range of immunological activities, including activating macrophages, DCs, and monocytes, inducing the synthesis of NO, regulating cell signal transmission related to immune response, minimizing the harm caused by ionizing radiation to the body’s immune system, and promoting the synthesis of IG. By binding pentatricopeptide repeat (PPR) of various immune cells, a series of cascade responses of immune defense is triggered, modifying both innate and adaptive immune responses, such as the key role of a group of (1→3)-β-glucans in regulating DC function. Further in-depth studies are required to understand the relationship between its various sources, complicated structure, and broad biological function. At present, the (1→3)-β-glucans have been widely employed in daily life as well as in the fields of medicine and biology. For instance, oral administration of (1→3)-β-glucans has been reported to lower levels of lipid in blood, regulate immune responses, and exhibit anti-tumor properties. They are also used as pharmaceutical and cosmetic ingredients, and so on. It is not expected to take much time, but it would show vast application prospects and huge application value. Regulating the human body’s immune response with (1→3)-β-glucans is promising to help improve the body’s immune status and provide new avenues for clinical anti-tumor, anti-infection, and anti-inflammatory treatment. With ongoing research into the immunological function and corresponding mechanism of action of the (1→3)-β-glucans, the functionally further evaluated (1→3)-β-glucans are expected to be employed more and more frequently.

## Figures and Tables

**Table 1:** Common β-glucans.

Linkage	(Glucan)	Source	References
**β-(1→2)**		*Mycoplasma capricolum* and *Mycoplasma leachii*	[26,27,28,29]
**β-(1→4)**	(cellulose)	Plant (common)	
**β-(1→6)**	(pustulan)	*Gyrophera esculenta*	[18]
β-(1→3)-**β-(1→4)**-β-(1→6)	(PSK)	*Trametes versicolor*	[30]
β-(1→6)-**β-(1→4)**	(LC11)	*Lentinus edodes*	[20]
β-(1→6)-**β-(1→4)**	(GLSA50-1B)	*Ganoderma lucidum*	[22]
**β-(1→6)**-β-(1→3)	(phytoalexins)	*P. megasperma mycelial*	[31]
β-(1→3)-glucans			
**β-(1→3)**	(pachymaran)	*Poria cocos*	[32]
**β-(1→3)**	(curdlan)	*Alcaligenes faecali*	[33]
**β-(1→3)**	(SSG)	*Sclerotinia sclerotiorum*	[34]
**β-(1→3)-β-(1→4)**	(hemicellulose)	Plant (common)	
**β-(1→3)-β-(1→4)**	(lichenan)	*Cetraria islandica*	[21]
**β-(1→3)-β-(1→4)**	(Barley glucan)	*Hordeum vulgare*	[33]
**β-(1→3)-β-(1→4)**	(Oat glucan)	*Avena sativa*	[15,16]
**β-(1→3)**-β-(1→6)	(schizophillan)	*Schizophyllum commune*	[35]
		*Candida albicans*	[36]
**β-(1→3)**-β-(1→6)	(lentinan)	*Lentinula edodes*	[37]
**β-(1→3)**-β-(1→6)	(Yeast glucan)	*Saccharomyces cerevisiae*	[11,12,13,14]
**β-(1→3)**-β-(1→6)	(scleroglucan)	*Sclerotium glucanicum*	[9,10]
**β-(1→3)**-β-(1→6)	(laminaran)	*Laminaria sigitota*	[9,10,38]
**β-(1→3)**-β-(1→6)	(grifolan)	*Grifola frondosa*	[39]
**β-(1→3)**-β-(1→6)	(pachyman)	*Poria cocos*	[32]
**β-(1→3)**-β-(1→6)	(PSGL-I-1A)	*Ganoderma lucidum*	[23]
**β-(1→3)-**β-(1→6)	(WGLP)	*Ganoderma lucidum*	[24]
**β-(1→3)**-β-(1→6)	(PGG)	*Saccharomyces cerevisiae*	[17]
**β-(1→3)**-β-(1→4)-β-(1→6)	(pleuran)	*Pleuritus ostreatus*	[19]
**β-(1→3)**-β-(1→6)	(WGP)	*Saccharomyces cerevisiae*	[25]
**β-(1→3)**-β-(1→6)	(zymosan)	*Saccharomyces cerevisiae*	[40]

**Bold linkages** mean a main chain in the glucan. SSG: β-(1→3)-D-glucan from *Sclerotinia sclerotiorum*. PSK: Polysaccharide K. PGG: poly-(1→6)-β-D-glucopyranosyl-(1→3)-β-D-glucopyranose glucan. WGP: Whole Glucan Particles. LC11: Branched (1→3;1→4)-β-glucan from *Lentinus edodes*.

**Table 2:** Clinical applications on (1→3)-β-glucans.

Study	Participants	Intervention	Main Findings	Ref.
Gudej et al. (2021)	129 participants with dyspepsia	Oat β-D-glucan Supplements	Reduced mucosal damage	[127]
Cheung et al. (2022)	One hundred two patients with HR-NB	Oral β-glucan Supplements	Elicited robust antibody responses in patients	[128]
Medeiros et al. (2012)	12 patients who had venous ulcers	Saccharomyces cerevisiae (1→3)-β-glucan Supplements	Enhanced venous ulcer healing and increased epithelial hyperplasia	[129]
Cicero et al. (2020)	83 Italian with a moderate hypercholesterolemia and a low cardiovascular risk	Oat β-glucan Supplements	Reducing LDL-C, TC and non-HDL-C	[130]
Bobovčák et al. (2010)	20 elite athletes	*Pleurotus ostreatus* β-glucan Supplements	May play a role in modulating exercise-induced changes in natural killer cell activity	[131]
Fuller et al. (2017)	49 participants	Yeast β-1,3/1,6 glucan Supplements	Prevent the occurrence or reduce the severity of upper respiratory tract infection	[132]
Fazilaty et al. (2018)	40 multiple trauma patients	β-glucan Supplements	Increase serum levels of IL-12	[133]
Lehne et al. (2006)	Eighteen healthy volunteers	Yeast β-1,3-D-glucan Supplements	Increased the immunoglobulin A	[134]
Auinger et al. (2013)	162 healthy participants	Yeast (1,3)-(1,6)- β-D-glucan Supplements	Increased the body’s potential to defend against invading pathogens	[135]
Lee et al. (2016)	30 patients in critically ill	β-glucan Supplements	Increases in natural killer (NK) cell activities	[136]
Carpenter et al. (2012)	60 recreationally active men and women	Yeast β-glucan supplementation	Stimulated cytokine production, increased total (CD14^+^)	[137]

## Data Availability

Not applicable.
